# Sensor-based proximity metrics for team research. A validation study across three organizational contexts

**DOI:** 10.3758/s13428-020-01444-x

**Published:** 2020-08-19

**Authors:** Jörg Müller, Julio Meneses, Anne Laure Humbert, Elisabeth Anna Guenther

**Affiliations:** 1 Universitat Oberta de Catalunya, Internet Interdisciplinary Institute IN3, Barcelona, Spain; 2 Oxford Brookes University, Oxford, UK; 3 Department of Management, Vienna University of Economics and Business, Vienna, Austria

**Keywords:** Wearable sensors, Bluetooth, Team science, Mixed-methods, Organizational context

## Abstract

Wearable sensors are becoming increasingly popular in organizational research. Although validation studies that examine sensor data in conjunction with established social and psychological constructs are becoming more frequent, they are usually limited for two reasons: first, most validation studies are carried out under laboratory settings. Only a handful of studies have been carried out in real-world organizational environments. Second, for those studies carried out in field settings, reported findings are derived from a single case only, thus seriously limiting the possibility of studying the influence of contextual factors on sensor-based measurements. This article presents a validation study of expressive and instrumental ties across nine relatively small R&D teams. The convergent validity of Bluetooth (BT) detections is reported for friendship and advice-seeking ties under three organizational contexts: research labs, private companies, and university-based teams. Results show that, in general, BT detections correlated strongly with self-reported measurements. However, the organizational context affects both the strength of the observed correlation and its direction. Whereas advice-seeking ties generally occur in close spatial proximity and are best identified in university environments, friendship relationships occur at a greater spatial distance, especially in research labs. We conclude with recommendations for fine-tuning the validity of sensor measurements by carefully examining the opportunities for organizational embedding in relation to the research question and collecting complementary data through mixed-method research designs.

## Introduction

Wearable sensors are providing exciting new research opportunities for the social sciences. Following up on initial technical developments to miniaturize and combine several sensor technologies into wearable devices in the first decade of the 21^st^ century, interested scholars have invested considerable effort in assessing the validity and reliability of the resulting data (Chaffin et al., 2017; Chen & Miller, 2017; Elmer et al., 2019; Kayhan et al., 2018). These initial studies relied mainly on laboratory experiments to assess the validity of sensors as indicators of physical constructs such as ‘proximity’ based on Bluetooth (BT) signals or ‘face-to-face’ detections based on infrared sensors. However, an increasing number of studies that deploy wearable sensors in real-world organizational settings have become available. This allows the variability of the sensor measurements to be assessed under realistic settings beyond controlled laboratory environments, while also putting the focus on the suitability of sensor data as indicators of higher-level social and psychological constructs. Several available studies have explored sensor data as indicators of “creativity” (Parker et al., 2018), “friendship” and “advice-seeking” (Matusik et al., 2018), or “subjective wellbeing” (Alshamsi et al., 2016) and “happiness” (Yano et al., 2015).

While studies based on real-world field settings make important contributions to assess the variability of sensor data and higher-level constructs, the existing variety of empirical field settings has been rather meager to date. Usually, wearable sensors are deployed in a single, relatively large group of people working together. The resulting findings are thus limited to one specific group and field situation without any means of extrapolating to other groups and/or conditions. This article addresses this problem by analyzing and comparing wearable sensor data among nine relatively small research and development (R&D) teams.

As a result, we therefore firstly offer important insights into the inter-group variability of Sociometric^1^ measurements for relatively similar R&D teams. By examining in more detail how important metrics vary between the nine comparable R&D groups, a more finely tuned picture of the context-sensitive nature of supposedly ‘objective’ sensor measurements begins to emerge. Secondly, our research also contributes to the important task of validating Sociometric, sensor-based measurements for higher-level constructs. BT signals are usually taken as a measurement of physical proximity between devices (or the people wearing them), which in turn should ideally provide a valid indicator of social ties such as friendship. Whereas others have shown that there is a moderate relationship between BT signals and these social ties, this article provides further insights into the strength of the relationship between Sociometric proximity and friendship on the one hand, and proximity and advice-seeking on the other, taking into account the different organizational embedding of groups. With regard to BT values, we can ask how reliably certain radio signal strength indicator thresholds discriminate between friendship or advice networks, not only within the same group but across several groups. By inserting important organizational and team-based control variables, we show how these thresholds might vary according to the wider context.

The article is structured as follows: in the first section we will briefly summarize the main findings of existing research using wearable sensors. This includes both laboratory validation studies as well as field research focusing on higher-level constructs. To the best of our knowledge, there has thus far been no study that analyzes sensor data across several comparable groups. Next, in the Methods section, the details of the field research are described in conjunction with the important data pre-processing steps carried out. In addition, there are also some initial sketches of the socio-demographic and sociometric profiles of the participating teams and an introduction to the overall analytical approach. The third section then describes the overall results, followed by a general discussion of their implications before we conclude the article with some final remarks and recommendations.

## Wearable sensor proximity measurements in organizational research

This article contributes to the validation of wearable sensors as a social science research instrument. Wearable sensors are one source of ‘big data’ that have attracted considerable attention from social scientists. Different sensor types provide access to previously hard-to-observe phenomena such as the heart-rate, small body movements and skin resistance, among many others (Foster, 2019; Ganster et al., 2018). Sensors gather high-resolution data on the often-unconscious bodily activity that underlies and to a large degree conditions the conscious social behavior that has been the traditional focus of social scientists. Alex ‘Sandy’ Pentland coined the term “honest signals” to describe how these semi-automated, subtle non-verbal cues are the basis for fluent communication and interaction (Pentland, 2008). The prospect of gaining access to a more fundamental layer of human behavior beneath the surface of words has led to intense research activity across the sciences. Computer scientists and engineers are developing artificial intelligence systems that ‘understand’ social situations by modeling, analyzing, and synthesizing non-verbal communication (Vinciarelli et al., 2009). Real-world applications are already in use to detect “deception” and “truth” (Elkins et al., 2012) in face-to-face situations, which in turn can be applied to decision-making on the “hireability” of candidates during job interviews (Chamorro-Premuzic et al., 2017; Nguyen et al., 2014).

Organizational researchers are exploring the possibilities of wearable sensors in the business context in order to optimize the productivity of employees and teams (Olguin et al., 2009; Pentland, 2012). Inspired by health scientists’ quest to track contact patterns and hence the spread of infections among people (Barrat et al., 2014; Duval et al., 2018; Fournet & Barrat, 2017; Salathé et al., 2010), social scientists have also studied the ‘spread’ or flow of information among team members (Fischbach et al., 2010; Kabo et al., 2015). Others have used sensor-based, high-resolution behavioral data for new types of time-based analysis of social phenomena such as leadership emergence (Cook & Meyer, 2017) or the ‘flow’ experiences of creative group interactions (Gaggioli et al., 2013; Parker et al., 2018).

Among the different types of sensor data available, this article focuses on measurements of physical proximity which are of special interest to organizational researchers. The importance of propinquity for social tie formation has been documented extensively across different spatial scales such as for example geographic regions, neighborhoods, organizations, or workspace layouts as well as for different types of social relations such as for example friendship ties, romantic ties, or knowledge exchange ties (Khazanchi et al., 2018; Rivera et al., 2010; Small & Adler, 2019). While the formation of social ties often involves face-to-face interaction in situations of physical proximity, conceptually social ties are not congruent with social interaction. Social ties, once established, can endure in time without actual interactions taking place. Friendship relations, for example, might be dormant during months but become activated in situations of crisis and when in need of support. Indeed, as Keyton (2018) argues, the frequency of interaction does not necessarily correlate with the perceived importance or influence of a social relation. Thus, while some have examined the impact of physical proximity on organizational behavior through direct face-to-face interactions (Boutellier et al., 2008; Elmer et al., 2019; Jeong and Choi, 2015; Vasileiadou & Vliegenthart, 2009), many publications in the field of social network research have shown that self-reported relational data provide an equally valid window upon key dimensions of organizational performance. In the context of organization studies, Kabo et al., (2014) have shown, for example, how proximity in terms of “shared paths to the lab” facilitate random encounters among researchers in the hallways, which increases not only the likelihood of (scientific) collaboration, but also the quality of the resulting work. More generally speaking, researchers are interested in the relationship between advice-seeking (instrumental) and friendship (expressive) ties with spatial proximity, as these two classes of social relationships affect knowledge-sharing behavior and ultimately team and overall organizational performance (Casciaro and Lobo, 2008; de Montjoye et al., 2014; Henttonen et al., 2010; Joshi & Knight, 2015). The ability to effectively address complex tasks hinges on having access to diverse knowledge resources (advice networks) on the one hand, and being able to integrate the available resources through social bonds (friendship networks) on the other (Kozlowski & Bell, 2003; Reagans & Zuckerman, 2001). Along these lines, wearable sensors offer interesting new research opportunities as they allow the gathering of highly granular proximity data among people. As such they provide new avenues to examine the link between propinquity and social tie strength, and its potential impact on organizational performance.

Hence the legitimate interest of Sekara and Lehmann (2014), for example, in assessing whether sensor proximity data correlate significantly with friendship ties (measured via social network ties on Facebook) among a group of 134 students. In an earlier study, Eagle et al., (2009) argued that by differentiating between a work and leisure context, smartphone proximity data can reliably identify friendship ties among a sample of college students. Similar findings have been reported by Oloritun et al. (2013, 25), who showed that the “duration (hours) of interactions, the period of interactions (e.g., weekday daytime, weekday night, weekend daytime and weekend night), floor similarities (residence) and gender similarities” predict friendship ties. The article by Matusik et al., (2018) should also be mentioned, since it estimates the convergent and discriminant validity of proximity data in a real-world, organizational setting. BT data collected over several weeks among team leaders in one large research facility suggested that friendship and advice-seeking/giving relationships correlate to some degree with BT proximity detections.

However, despite these initial encouraging results on the potential of using sensor-based proximity data as indicators of instrumental and expressive social ties, the results are limited for several reasons. Firstly, and most importantly, little is known about the influence of organizational and other contextual factors on sensor measurements. Despite Johns’ (2006) call to recognize the impact of context on organizational behavior, most research using wearable sensors brackets together broader, environmental variables from its analysis. This is partly to do with the fact that sensors enjoy an aura of ‘objective’ measurement whose validity transcends the specificity of time and place (Chancellor et al., 2017). It is also related to the fact that sensors measure “honest signals”, that is, behavioral cues that have been singled out as strong contextual markers for social interaction themselves (Goodwin and Duranti, 1992). As a consequence, organizational context has been largely ignored in (wearable) sensor research, despite the recognition that “strong cultural norms” considerably facilitate the identification of behavioral patterns such as “friendship” (Eagle et al.,, 2009, p. 15275). This article fills this gap by presenting an explicit comparative research design, collecting wearable sensor data from nine research & development (R&D) teams across the UK and Spain. R&D teams work under circumstances of high task complexity which requires the integration of diverse resources (knowledge, skills, etc.). At the same time, the wider organizational context might influence their collaborative activities and hence the micro-processes captured with sensors. To the best of our knowledge, our study is the first to comparatively examine the influence of organizational factors on the validity of BT-based proximity measures as indicator of (self-reported) social ties.

A second limitation of existing research concerns the relative scarcity of validation studies carried out in real-world organizational settings. Different validation studies of wearable sensors have been carried out as laboratory experiments (Chaffin et al., 2017; Kayhan et al., 2018), whereas real-world settings usually involve interaction patterns among college students (Blok et al., 2017; de Montjoye et al., 2014; Dissing et al., 2018; Eagle et al., 2009). Explicit validation studies targeting instrumental and expressive ties in real-world organizational settings, however, are relatively scarce. Although there are studies that examine wearable sensors in relation to nurses’ “workload” (Yu et al., 2016), “affect dynamics” (Alshamsi et al., 2016) or compare Sociometric badges versus smartphone-based proximity measurements (Boonstra et al., 2017), only Matusik et al., (2018) has a more detailed interest in organizational research, as already mentioned. Our study contributes to this emerging field by providing further evidence of the validity of wearable BT detections for friendship and advice-seeking behavior in real-world settings. Interested researchers will gain insights into the relative validity of wearable sensors for measuring these important dimensions of expressive and instrumental relationships in work groups. We conceive this as a further preparatory step in order to unlock the true potential of wearable sensor data in organizational research, namely to advance in the time-based analysis of the formation, duration, frequencies, and cyclicality of social relationships. The high-resolution temporal data gathered with wearable sensors will be crucial for the analysis of the dynamic nature of many social phenomena (Lehmann-Willenbrock et al., 2017).

## Methods

### Field access and data collection

Eight R&D teams were recruited in autumn 2016 and spring 2017 in Spain and the UK. Three of our research groups operate from within public universities (T1, T3, T6), while three groups work in public research centers (T2, T4, T5) and three more teams belong to a private company (T7.1, T7.2 and T8; Team 7 is one team in two different locations within the same company, which we effectively count as two teams given the two distinct field periods, separate working environments and lack of face-to-face meetings during the observational period). While the R&D teams in the private company work in the construction sector, all others work in the field of biomedical engineering. This provides a total of nine R&D teams comprising 80 individuals.

The same field-work procedure was carried out for each team. Data were collected for five consecutive working days using Sociometric badges (Humanyze, Boston, USA; formerly Sociometric Solutions) with the team. At the start of the fieldwork, a short introduction was given to all team members on how to wear the badge, how to turn it on/off and where to pick it up in the morning and drop it off in the afternoon or whenever people left work. Team members were also instructed to note any exceptional occurrences when the badges were turned off or people were absent from work.

A short online questionnaire was sent to each team member during or shortly after the actual fieldwork with the Sociometric badges, in order to collect the socio-demographic variables of each member, as well as additional information on certain team characteristics that would allow the results of the sociometric profile to be contrasted and interpreted.

Each participant indicated their friendship and advice-seeking ties with each of their colleagues. First, team members were asked to indicate the frequency with which they ask each of their colleagues for ‘work-related advice’ (1=Never, 2=Rarely, 3=Sometimes, 4=Very often, 5=Always). We used a factual question on advice-seeking as others have done in social network studies (Brennecke and Rank, 2016; Lazega et al., 2012), but introduced a more detailed response scale in terms of frequency of consultation. Given the small team size, advice-seeking behavior is to be expected and would discriminate little when using a binary response (yes/no) over a fixed time period (De Lange et al., 2004). Descriptive statistics of self-reported advice-seeking scores are available in the supplementary file S1—Table 1 and S3—Illustration 1B.

In response to a second round-robin question, participants indicated the frequency with which they ‘spend time socially’ with each of their colleagues outside the lab/office (1=Never, 2=A few times a year, 3=A few times a month, 4=A few times a week, 5=Daily). The resulting scores can be interpreted as indicating that the relative strength of a friendship relationship based on the frequency of spending off-work time together. Following previous sensor/proximity studies (Eagle et al., 2009; Wuchty, 2009), we use a behavior-based (‘spending time together’) rather than a belief-based (‘who do you consider your friend’) indicator of friendship, as this enables a more precise definition of ‘friendship’ ties in our case studies. The descriptive statistics of self-reported friendship scores are available in supplementary file S1—Table 1 and S3—Illustration 1A.

As the existing social network literature has shown, self-reported measures, although not free from bias, provide a sufficiently reliable method to measure expressive and instrumental ties against which BT detections can be assessed (see De Lange et al.,, 2004).

This research was approved by the Ethics Committee of the Universitat Oberta de Catalunya and the Ethics Review Process of the European Commission. Participants signed separate Informed Consent sheets for wearable sensor data collection and semi-structured interviews. The online questionnaire displayed information on data and privacy protection before it began. Each participant was also offered the possibility of wearing a ‘dummy’ Sociometric badge instead of a fully functional device in a private email exchange to prevent non-participants from being singled out or subject to peer pressure. Although in some cases team members declined to wear badges, no ‘dummy’ badge was used at any point of the research.

### Data pre-processing

The analysis of sociometric data involves several pre-processing steps after the captured data has been downloaded from Sociometric badges to the computer. This includes the selection of export parameters for the ‘raw’ data, and the formatting, clean-up, and aggregation of some variables before they are made available in the actual analysis. The firmware settings of the Sociometric devices are provided as supplementary material (file S2). All data pre-processing and data analysis was carried out using the R software environment for statistical computing; scripts are available as supplementary files—S4. The original data set has been published as R software package by Müller et al. (2018), including documentation on data clean-up steps and scripts.

#### Bluetooth RSSI range

Sociometric badges are equipped with a Bluetooth sensor. Mutual BT detections are registered as numeric scores, through the so-called Radio Signal Strength Indicator (RSSI). Each Sociometric badge scans autonomously for other devices in its vicinity roughly every 25 s while scans last approximately 10 s during which the scanning device itself cannot be discovered. The scanning cycles of badges are not synchronized among devices. If device A detects another device B, the corresponding RSSI score is recorded on the internal memory of device A together with a timestamp (hour, minute, second) and the ID of device B. The smallest interval between consecutive detections can be below 25 s, for example when device A has just finished a scan and becomes discoverable as device B starts its scan. As a result, it is not uncommon to have a rapid series of mutual detections among Sociometric badges while devices are in proximity to each other.

RSSI values usually range from -90 to -40, where higher numbers such as -40 indicate a stronger signal which is usually produced by devices being closer together (Liu & Striegel, 2011). Although stronger RSSI values (e.g., -40) usually indicate devices being closer together while weaker signals (e.g., -90) indicate greater spatial separation, other factors such as cubicle walls, clothing worn over the device or the angle in which devices are situated can affect RSSI scores independent of physical distance (Chaffin et al.,, 2017; Müller, 2018, p. 26). RSSI scores also exhibit some variance under stable experimental conditions: at a fixed distance of 1.4 m between devices, the registered RSSI values cover almost the entire range of theoretically possible values (minimum = -90, maximum = -50, with mean RSSI = -70.63, standard deviation = 7.45, *N* = 516 detections, see Müller et al.,, 2018). Variations in the physical distance between devices will produce corresponding increases or decreases in the mean RSSI value. Importantly, BT detections do not necessarily indicate face-to-face interactions, as detections can occur within up to 10 m of distance (see Chaffin et al.,, 2017). Taking all these factors together suggests that decreasing RSSI levels, although influenced by increasing spatial distance, cannot be translated into equidistant increments where each RSSI level would correspond to a fixed distance of 10 cm, for example. We therefore interpret the RSSI values in terms of closer spatial proximity or greater spatial distance similar to Matusik et al., (2018), but avoid references to precise measures of physical distance.

**Fig. 1:**
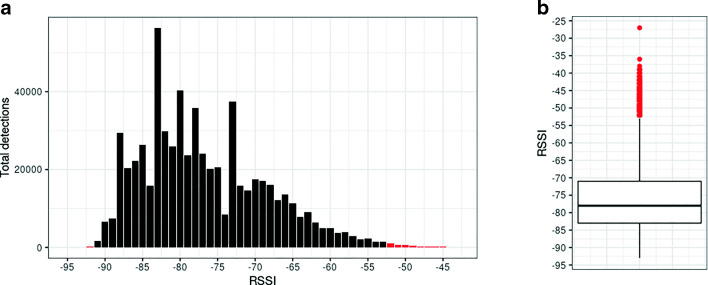
Distribution **a** of BT detections by RSSI level across all teams. Removed RSSI levels are shown in red. **b** Boxplot showing IRQ of BT detections with median (-78)

In our current data set, RSSI values range from -93 at the lower bound to a maximum value of -27 at the upper bound, suggesting that some detections occur at extremely strong or weak RSSI levels as outliers. BT detections occurring at these extreme values should be examined and possibly removed from the analysis, either because the signal is too weak (and thus participants too distant to be interacting) or too strong (probably a measurement error) to be counted as real co-presence. In order to remove spurious detections, we identify outliers based on the interquartile range (IQR) of the collected data, which ranges from -83 to -71, with a median RSSI value of -78 for all nine teams. Outliers below the third quartile -1.5 x IQR and above the first quartile + 1.5 x IQR are BT detections that occur at weaker RSSI levels than -101 and stronger RSSI levels than -53, respectively. No outliers exist at the lower bound. However, in order to guarantee a sufficient number of team member dyads (*n*> 30) for our analysis, we removed BT detections at the lower bound occurring at RSSI < -91. At the upper bound, we removed outliers above RSSI > -53. The minimum number of team member dyads observed is thus *n* = 39 at RSSI = -91 and *n* = 32 at RSSI = -53. According to Bonett and Wright (2000), we deem this a sufficient number of cases for our analysis of correlations. Given that we have a total of 625,428 detections across nine teams, removing 3829 detections (shown in red in Fig. 1) yields a total of 621,599 detections to be included in our analysis.

#### Interpolating self-reported friendship and advice-seeking scores

In order to examine the correlations between BT detections and self-reported measures, several pre-processing steps have been carried out. First, self-reported ratings are directional, in that person A rates person B on the two available scales, including frequency of ‘work-related advice’ and frequency of ‘spending time socially’ outside the office. BT detections on the other hand are not directional and are recorded for team member pairs. Thus, although we have two directional self-reported ratings, we only have a single BT detection figure for each team member dyad. This implies that two self-reported ratings per dyad need to be matched to the single number of BT detections for the same team member dyad. We thus convert the directional self-reported ratings into a single friendship and advice-seeking score for each team member dyad. Depending on the rating in question, we have deployed slightly different rationales, which are explained below.

First, the frequency with which team members ask each other for work-related advice was scored on a scale from 1 to 5. The frequency with which person A asks person B for advice may be independent of how often person B asks person A. For example, students would be expected to ask senior team members for advice more often, but this does not imply that seniors ask junior members equally often. Following this logic, we stipulated that the overall advice-seeking score is the sum of ‘score A + score B’. Person A ‘rarely’ (score 2) asking B, while B ‘always’ (score 5) asks A, would result in ‘2 + 5 = 7’.

Second, spending time socially with each of their colleagues outside the lab/office is different from the advice scores, since the rating should refer to the same empirical situation. If person A indicates that they meet person B ‘daily’ outside of work but person B indicates that they only meet ‘a few times a month’, the statements are contradictory in terms of only one possible empirical reality. Given that people’s perception of their friendship ties are ego-centered and thus coincide only poorly with each other (Almaatouq et al., 2016), we take the mean value of both scores, assuming that the best available estimation of the strength of the relationship lies in-between those individual scores. Hence, the mutual friendship score is calculated by the ‘sum(score A + score B) / 2’.

Missing friendship and advice-seeking scores have been treated using the same method: if only one rating is available per team member dyad (A rates B but B does not rate A), we replace the corresponding NA entry with the existing score. This assumes that alter would rate ego in the same way that ego rated alter. Out of the 614 possible ratings among the respective team members we replaced 62 (10,0%) missing friendship ratings and 57 (9.3%) missing advice-seeking ratings of ego with the corresponding existing scores of alter. If both entries are missing, the dyad is dropped from the analysis. Across all nine teams we have 307 possible dyad ratings. Two dyads were excluded from friendship ratings and one dyad from advice-seeking ratings due to missing values for both ego and alter entries, leaving us with 305 valid friendship ratings and 306 valid advice-seeking ratings.

Since we use a linear transformation in both cases where the self-reported scores are interpolated, its effect on the magnitude of the correlation coefficient is negligible. However, the chosen transformations are nevertheless carried out following the other examples available in research (Van der Vegt et al., 2010), in order to achieve a more straightforward interpretation of the self-reported measures.

### Analytical approach

The overarching question addressed by our research concerns the validity of Bluetooth signals as indicators of social ties. Given the importance of propinquity for social tie formation as argued previously (Eagle et al., 2009; Onnela et al., 2014; Wuchty, 2009), we further contribute to this second strand of the literature by examining the correlation between BT signals (as a measure of physical distance) and self-reported social ties. In other words, instead of examining to which degree BT signals capture social interaction, we set out to further examine to which degree BT detections reliably capture expressive and instrumental ties as important dimensions of organizational behavior. Our analytic approach for answering this question is outlined in the following paragraphs.

#### Validity of cumulative BT detections

Before we can take full advantage of sensor-generated interaction data, it is important to clarify the extent to which BT detections converge with established measurements in organizational research. We thus explore the convergent validity of BT detections with relational data by calculating the correlation coefficient between the frequency of BT detections and the corresponding self-reported score for friendship on the one hand, and the score for advice-seeking on the other. In order to do this, we assign a unique ID to each pair of interaction partners, effectively pooling all team member dyads across all teams. We then calculate the self-reported scores for advice-seeking and friendship for these team member dyads as described previously. In a further step, we count the total number of BT detections for each member dyad across an increasing range of RSSI levels. Thus, the first RSSI interval is defined at -53 only (the strongest RSSI level, indicating closer spatial proximity), a second interval from -53 to -59, a third from -53 to -60 and so on, until BT detections occurring across the entire range of RSSI levels from -53 to -91 (indicating greater spatial separation) are counted. Starting from stronger RSSI signals and incorporating more and more liberal RSSI levels assumes that BT detections occurring at stronger RSSI levels (indicating closer spatial proximity) are more indicative of specific social ties compared to BT detections occurring at weaker RSSI levels. Incorporating BT detections at weaker RSSI levels are likely to be less indicative of specific social ties since detections at greater spatial distance tend to capture a wider variety of social situations, including purely random co-locations. For each interval, we add together the BT detections occurring at the included RSSI levels while ignoring BT detections falling outside of it. Calculating the cumulative BT detections at increasingly wider RSSI intervals will provide an indication of the range of RSSI levels at which BT detections best converge with self-reported social relations. We therefore provide an important contrast to many studies using wearable sensors that chose the RSSI threshold for cumulative BT detections more or less arbitrarily. Finnerty et al., (2014) for example, use a RSSI range of [-80, -60] and [-85, -80] to distinguish between close range (less than 1 m) and intermediate proximity (from one to 3 m) without systematically testing more inclusive ranges.

In a final step, we calculate the Spearman’s rho (r_s_) correlation coefficient between BT detections (count data) and reported friendship scores (ordinal data) on the one hand, and BT detections and reported advice-seeking scores (ordinal data) on the other. This yields a series of correlation coefficients, one for each of the defined RSSI intervals. Thus, the first correlation coefficient is calculated between BT detections occurring at -53 indicating close spatial proximity, and a second covering BT detections occurring within a more liberal RSSI range from -53 to -54, all the way down to the correlation between BT detections occurring over the entire RSSI range from -53 to -91. Overall, we expect that team member dyads that indicate stronger advice-seeking ties or stronger friendship ties will be found more frequently in the vicinity of each other and therefore produce higher BT detections versus team members that score lower on the respective self-reported measures. As a result, strong correlation coefficients (i.e., close to 1) indicate that higher BT detections tend to coincide with higher self-reported scores and lower BT counts with lower scores. A negative correlation coefficient (i.e., close to -1) implies that higher BT counts tend to coincide with lower self-reported scores. Low correlation coefficients (i.e., close to 0) imply that there is no consistent relationship between the magnitudes of BT detections and social relationship scores for a given RSSI level, i.e., high BT counts are associated with both, low and high self-reported scores. For a schematic account how strong versus low correlation coefficients can be generated by different configurations of BT detections in combination with varying tie strength of self-reported measures, see supplementary file S3—Illustration 2.

Following Hemphill (2003), who reviewed 380 meta-analytic studies in psychological sciences, we consider a correlation coefficient below r <.20 as weak, from .20 to .30 as moderate and r >.30 as strong. Our usage of r >.30 as upper threshold for strong correlation coefficients contrasts with the suggested larger, upper threshold of r >.50 as originally stipulated by Cohen (1988). However, effect sizes larger than r >.50 do occur rather infrequently in most psychological research as pointed out by Hemphill (2003), which suggests that r >.30 should be considered as a threshold for strong correlations.

#### Validity of BT detections at discrete RSSI levels

The cumulative use of BT detections provides insights into the extent to which BT detections coincide with self-reported social relations, i.e., how well sensor measurements capture self-reported friendship and advice–seeking, irrespective of spatial distance. The use of BT detections at discrete RSSI levels shifts the focus towards exploring the role of spatial proximity in distinguishing between friends and non-friends or advice-seeking and non-advice-seeking relationships. As Hall (1992) observed, the minimum distance which is tolerated among interaction partners varies according to the type of social relation concerned and ranges from intimate relations involving touching to more informal settings up to 4 m. We therefore stipulate that different social relationships correspond to different patterns of spatial proximity between people—as measured by the Bluetooth radio signal strength (RSSI). In the case of friendship for example, it seems plausible that friends rather than non-friends communicate more often and for that purpose meet more frequently in close physical proximity to each other, thus increasing BT detections at higher RSSI values. Advice-seeking relationships on the other hand operate on a more formal level, which could translate into a greater spatial separation among team members and in turn produce higher BT detections at lower RSSI values (Matusik et al., 2018). This suggests the need to examine for each discrete RSSI level the correlation between the frequency of BT detections and the friendship score on the one hand, and BT detections and the advice-seeking score on the other. We want to understand the specific RSSI level at which we obtain the strongest correlation between BT detections and self-reported scores.

In order to examine the validity of the BT detections at discrete RSSI levels, instead of counting all detections across the entire RSSI range from -91 to -53, we therefore bin the absolute counts for each level. We count the detections for each interaction dyad at the specified RSSI value (e.g., -70) while ignoring all other interactions that occur at stronger signals (e.g., -69, -68, ..., -53) or weaker signals (e.g., -91, -90, ..., -71). Using the unique ID of each team dyad, its BT count is associated with the corresponding self-reported scores for friendship and advice-seeking, respectively. Given the association of friendship and advice-seeking scores with the characteristic BT detections of each team member dyad, we then calculate the corresponding Spearman’s rho (r_s_) correlation coefficient for a given RSSI level.

#### Contrast of BT statistics between high and low self-report measures

In order to better interpret the relationship between the frequency of BT detections at discrete RSSI levels and hence spatial distance, we furthermore assign team member dyads to a ‘strong’ versus ‘weak’ friendship group and a ‘strong’ versus ‘weak’ advice-seeking group, respectively. Team member dyads belong to the group with strong friendship ties if their friendship score falls into the 4^th^ quartile (i.e., 25% of highest) of these self-reported scores within their team. They belong to the group with weak friendship ties if their friendship score falls into the 1^st^ quartile (i.e., 25% of weakest) of these self-reported scores within their team. The same holds for the respective high versus low advice-seeking groups. The summary statistics of strong/weak friendship and advice-seeking groups is available in Table 1 (see also the corresponding boxplot in supplementary file S3—Illustration 3). We then retain only those BT detections of team member dyads that belong to either of these groups while BT detections of all other dyads are ignored. We expect that BT detections among team members belonging to the strong friendship group tend to be higher and tend to occur at a stronger RSSI level than BT detections among team members belonging to the weak friendship group. The same holds for dyads having strong versus weak advice-seeking ties.

#### Assessing the influence of the organizational context

As an important contribution to existing research, we explore how the organizational embedding of the participating research groups affects the validity of cumulative BT detections on the one hand and the validity of discrete RSSI measures on the other. As mentioned previously, three research teams work in public universities, while three teams operate in public research centers and three more teams belong to a private company. These differences are important because organizational context has been shown to affect teams in a variety of dimensions ranging from research performance (Baird, 1986; Bland and Ruffin, 1992; Bonaccorsi & Secondi, 2017; Verbree et al., 2015) and creativity (Heinze et al., 2009) to the impact of diversity (Joshi & Roh, 2009) and learning (Zellmer-Bruhn & Gibson, 2006), among others.

**Fig. 2:**
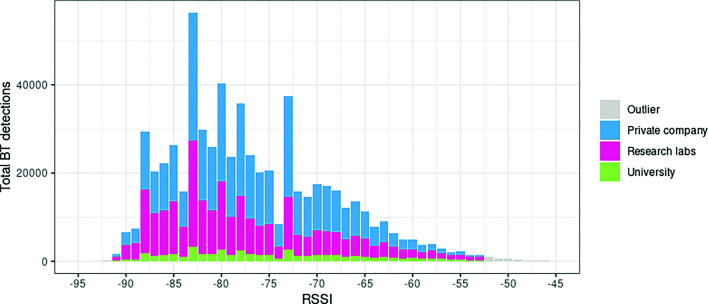
Stacked bar plot of absolute BT detections across organizational contexts

Differences between organizational missions seem to affect the frequency of communication and the intensity of interaction as shown in Fig. 2. Clearly visible is the much smaller number of BT detections taking place among team members working in the university context compared to the private company or the research labs. Academics who work in universities have to divide their time between teaching responsibilities as well as research. Depending on the amount of credits taught, the availability of team members to each other is naturally limited, because they are ‘in class’, ‘tutoring students’, ‘participating in committees for MA or PhD defenses’, etc.

At the same time, as our own observations made clear, individual office space for single researchers was more readily available for staff working in universities than those working in the research labs or the private company (for a schematic illustration of the work-space layout in universities, see supplementary file S3—Illustration 4). In contrast, team members working in research centers or the private company are usually available to each other to a higher degree because they share office or laboratory space on a more continuous basis. A crucial difference between the laboratory environments and the office space of the private company participating in our study, concerns the relative mobility of workers: in research centers, staff usually moves between several functional workspaces within the laboratory, including their desks and experimental facilities such as microscopes, wet benches or fume hoods (see S3—Illustration 5). Team members working in the private company on the other hand, carry out mostly computer-based work such as programming, virtual meetings, or design activities. Hence, they work at their desktop stations, relatively immobile and at a fixed distance to each other due to their seating layout (see S3—Illustration 6). A quick glance at the average interactions per team member and hour confirms the organizational difference between working environments: team member dyads in universities have on average 15 detections per hour, whereas those in research labs have 44 detections and members of company teams generate 88 detections per hour on average (see Table 1). These observed differences in terms of BT detection frequencies mirror similar findings observed in the literature on traditional versus open and shared workspace environments. Open office spaces facilitate more frequent and shorter face-to-face interactions whereas interactions are less frequent in traditional environments with cellular and more separated workspaces (Elsbach & Pratt, 2007; Khazanchi et al., 2018).

**Table 1:** Comparison of selected BT statistics between team member dyads scoring in the 1^st^ quartile and the 4^th^ quartile of self-reported friendship and advice-seeking scores

Organization	Selected dyads	Absolute BT detections	Mean BT by dyad	Mean BT by dyad and hour	Mean RSSI	Median RSSI
All teams	Strong advice ties	121830	2437	61	-74.76	-76
All teams	Weak advice ties	61126	1609	40	-78.20	-80
All teams	Strong friendship ties	105996	2208	55	-77.07	-78
All teams	Weak friendship ties	30769	1140	28	-78.18	-80
University	All dyads	47512	617	15	-74.72	-76
University	Strong advice ties	12165	760	19	-71.99	-72
University	Weak advice ties	1607	179	4	-83.36	-84
University	Strong friendship ties	10609	558	14	-73.42	-74
University	Weak friendship ties	641	214	5	-73.38	-73
Research labs	All dyads	237311	1745	44	-77.73	-80
Research labs	Strong advice ties	51577	2242	56	-75.12	-77
Research labs	Weak advice ties	32632	1554	39	-78.23	-80
Research labs	Strong friendship ties	57340	2606	65	-78.49	-80
Research labs	Weak friendship ties	15297	900	22	-76.73	-79
Private company	All dyads	336776	3508	88	-76.74	-78
Private company	Strong advice ties	58088	5281	132	-75.01	-76
Private company	Weak advice ties	26887	3361	84	-77.86	-79
Private company	Strong friendship ties	38047	5435	136	-75.95	-77
Private company	Weak friendship ties	14831	2119	53	-79.88	-81

Corresponding BT detects of strong versus weak self-reported measures are counted across all RSSI levels from -91 to -53. Mean BT by dyad is based on the number of observed dyads only (not all possible team member combinations). Mean BT detections by hour are based on a total of 8 working hours over 5 days (40 h)

Given the differences in interaction opportunities between university-based teams vs. research labs and private company-based teams, we expect to observe differences in terms of the correlations between BT detections and self-reported measures according to the different organizational contexts. We thus subdivide our interaction pool according to the three-fold organizational setting of university, research labs and private company. For each organizational group we then pool all interactions and re-run our cumulative and discrete analysis and examine the extent to which we can observe significant differences between organizational contexts for the same type of relationship.

In order to determine whether observed differences between organizational contexts are significant, a simple visual inspection of the different correlation curves is not enough. It is also insufficient to interpret a statistically significant correlation in one group (e.g., friendship in university teams) and the absence of a statistically significant correlation in the other group (e.g., friendship in research labs) as evidence of a statistically significant difference between both groups. Hence, in order to assess whether the correlation coefficients of BT with self-reported measures are significantly different between the three types of organizations, the ‘cocor’ R software package was used. As detailed in the paper by Diedenhofen and Musch (2015), the cocor package provides a robust statistical test for the comparison of the magnitude of two correlations. Based on our results, we can identify for which discrete RSSI levels the difference between the correlation coefficients of ‘university vs. research labs’, ‘university vs. private company’ and ‘research lab vs. private company’ is significant.

#### Test of independence between friendship and advice ratings

Assessing the validity of sensor-based measurements is dependent on the validity of our self-reported measurements. In a further pre-processing step, the discriminant validity of our two measurements on friendship and advice-seeking relations within each team has been tested. Some studies suggest that social relationships based on affection or social affinity have an influence on task/skill-related ties (Casciaro & Lobo, 2008). In order to avoid confounding the two types of self-reported measures during the correlation analysis, we test for sufficient difference between the instrumental (advice) and expressive (friendship) scores by applying a Quadratic Assignment Procedure (QAP) (Borgatti et al., 2013; De Lange et al., 2004; Krackhardt, 1988) available in the sna R package (Butts, 2019).

QAP is a non-parametric technique suitable for social network data where the assumption of independence between observations does not hold. The model tests for statistically significant correlations based on random permutations of the network data. The QAP permutation test has been performed on the self-reported measures (1000 iterations). The mean correlation between friendship and advice-seeking scores across all teams is r = .23. Correlation coefficients range from r = -.057 (Team 6) to r = .391 (Team 4) and r = .399 (Team 1). Only the correlation coefficient of Team 1 and Team 4 are significant at the p <.05 level (see S1—Table 2). Overall, self-reported friendship and advice-seeking ties only correlate moderately or weakly, similar to findings reported in Matusik et al., (2018). We conclude that friendship and advice-seeking networks are sufficiently different from each other to discriminate between expressive and instrumental ties.

#### Reference distributions for correlation coefficients

In order to assess the significance of the correlation coefficients between observed BT detections and self-reported measures, several reference distributions have been generated using the QAP (Brusco and Steinley, 2015; Hubert & Arabie, 1989). These simulated reference distributions provide a benchmark for the likelihood to obtain the observed correlation coefficients between BT detections and self-reported measures by chance only. Following the logic of the QAP, the columns and rows of each of the self-reported measures are reshuffled at random before calculating the correlation coefficient with the corresponding matrix of BT detections which is held constant (all RSSI levels are included). Overall, 1,000 matrix permutations have been carried out and the corresponding correlation coefficients with BT detections calculated. A comparison of simulated reference distributions with the empirical distribution of the observed correlation coefficients across organizational contexts is available in supplementary file S3 - Illustrations 7 to 10. The .5%, 2.5%, 97.5% and 99.5% quantiles for simulated reference distributions are reported in supplementary file S1 - Table 3.

## Results—validity of Bluetooth proximity metrics

As the following section will demonstrate, there are significant differences not only between the peak correlations of each type of social relationship across all teams, but also between organizational sub-groups. In addition, there are significant differences for each of the examined self-reported measures when comparing clusters of teams with each other.

### Validity of cumulative BT detections

**Fig. 3:**
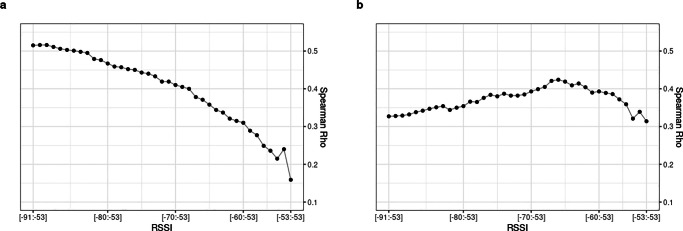
Correlation coefficients for **a** friendship and **b** advice-seeking scores and cumulative BT detections at increasingly wider RSSI intervals across all teams

Figure 3 shows Spearman’s rho correlation coefficient for BT detections with (A) friendship scores and (B) advice-seeking scores (see Table 4 and 5 respectively in the supplementary file S1). Using data from all teams, cumulative BT detections converge with self-reported friendship scores the wider the interval of RSSI values included. A linear relationship can be observed in Fig. 3a which shows that the less inclusive the RSSI levels for which BT detections are counted, the weaker the correlation coefficients. The correlation is strongest at r_s_ =.52 when BT detections across the entire range of RSSI levels from -91 to -53 are included. Excluding RSSI levels and thus constructing more narrower RSSI ranges produces weaker and weaker correlations with friendship scores. The lowest correlation coefficient is obtained for BT detections at the strongest RSSI signal of -53. Given that for psychological research a correlation coefficient of r >.3 can be considered strong (Hemphill, 2003), we conclude that BT detections and friendship scores do converge. Although the maximum correlation coefficient of r_s_ =.52 can be considered strong, the amount of variance explained is still quite limited at 27%. Comparing the group of team members that report strong friendship ties (4^th^ quartile) versus weak friendship ties (1^st^ quartile) indicates that ‘friends’ generate on average 55 BT detections per hour versus only 28 BT detections of ‘non-friends’ (see Table 1). The chance to obtain a correlation coefficient as high as r_s_= .52 tends towards 0, given that correlation coefficients above r_s_ >.46 occur in less than .5% of all random simulations (see S1—Table 3).

The results differ considerably for advice-seeking relationships, as can be seen in Fig. 3b. Correlation coefficients are now at their lowest when BT detections across the entire RSSI range are counted. As weaker RSSI levels are excluded, the correlation coefficient increases in a linear fashion until it reaches a peak correlation of r_s_ =.42, counting only BT detections that fall within a relatively narrow RSSI interval from -66 to -53. Using BT detections in an even narrower RSSI range again yields weaker correlations. In contrast to cumulative friendship scores which produces the strongest correlation coefficient when including BT detections across the entire RSSI range, for advice-seeking this logic does not hold. A more selective approach which limits the BT counts to higher RSSI levels—indicating closer spatial proximity—produces stronger correlation coefficients with advice-seeking scores up to a certain point. The peak correlation of r_s_ =.42 is still strong according to Hemphill (2003), suggesting that advice-seeking scores and BT detections converge, although to a lesser extent than the correlation coefficient with friendship scores. The variance explained for the peak correlation of r_s_ =.42 in the case of advice-seeking relationships is therefore 18%. Dyads that self-report strong advice-seeking ties (4^th^ quartile) generate on average 61 BT detections per hour versus only 40 BT detections of team members that self-report weak advice-seeking ties (1^st^ quartile, see Table 1). The chance to obtain a correlation coefficient as high as r_s_= .42 tends towards 0, given that correlation coefficients above r_s_ > .28 occur in less than .5% of all random simulations (see S1—Table 3).

#### Validity of cumulative BT detections and organizational context

When examining the validity of cumulative BT detections with social relationships across the three types of organizational contexts, marked differences become apparent, particularly for university-based teams (see Table 4 and 5 respectively in the supplementary file S1).

**Fig. 4:**
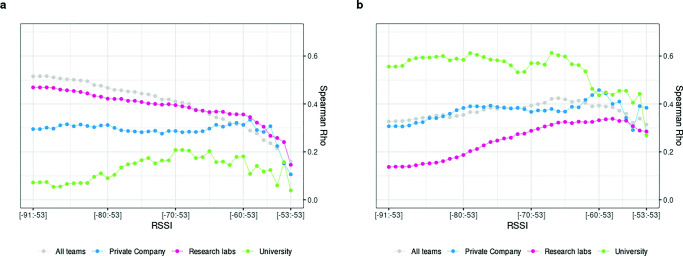
Convergent validity of **a** friendship and **b** advice-seeking scores with cumulative BT detections by organizational context

Figure 4a compares the correlation of BT detections with friendship scores between organizational contexts. BT detections continue to converge strongly with friendship scores when wider RSSI intervals are considered for research labs (maximum r_s_ =.47 with .5% of simulated cases above r_s_ >.33). The private company shows a rather stable correlation coefficient of around r_s_ =.30, independent of the chosen RSSI range (maximum r_s_ =.33 at [-53:-59], with 2.5% of simulated cases above r_s_ >.35). The behavior of university-based teams is however markedly different, with correlation coefficients being lower overall and showing an inverted tendency: the maximum r_s_ =.21 is reached at a more restrictive range of RSSI values, namely at [-53:-69], not being significant since 2.5% of simulated cases are above r_s_ >.34. University-based teams thus invert the overall tendency to produce stronger validity the wider the RSSI interval over which BT detections are counted. This suggests that for research labs, friendship ties are better captured using all RSSI levels, whereas for university-based teams, a more restricted interval should be used. Given the peak correlation coefficients for each type of organization, we can conclude that friendship ties converge strongly (r_s_ >.30) with BT detections for research labs and the private company, whereas they only converge moderately (r_s_ <.30) for university-based teams. In addition, team member dyads that self-report strong friendship ties (4^th^quartile) generate on average more BT detections per hour compared to dyads reporting weak friendship ties (1^st^ quartile). The corresponding average BT detections per hour for strong/weak self-reported friendship ties are 14/5 for university-based team member dyads, 65/22 for research labs, and 136/53 for company-based teams (see Table 1).

**Fig. 5:**
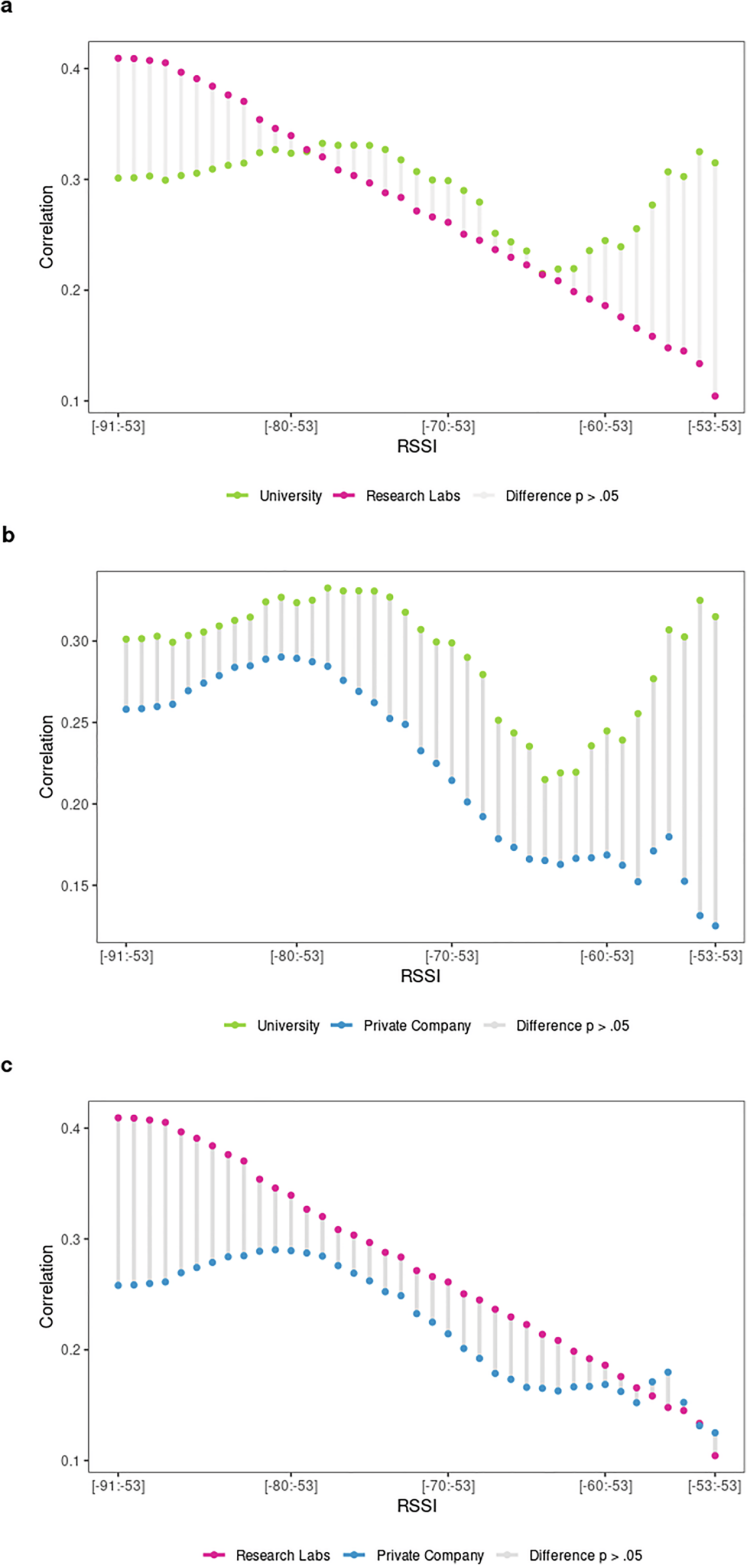
Differences of friendship correlation coefficients at cumulative RSSI intervals comparing organizational contexts

However, as the cocor test suggests, differences between organizational contexts are not significant. Comparing the correlation coefficients of the same social relationship between independent organizational groups (university versus research labs, university versus private company, and private company versus research labs) does not provide any significant results (see Fig. 5 and Table 8, 9, 10, respectively in the supplementary file S1). This means that the observed differences in terms of convergence between friendship scores and BT detections, according to the three organizational contexts, could have occurred by chance.

Turning to advice-seeking next, the correlation coefficients display some interesting changes, again especially for university-based teams as shown in Fig. 4b (see Table 5 in the supplementary file S1). Overall, the magnitude of the correlation has clearly increased for university-based teams, reaching a correlation coefficient of r_s_ >.60 for some intervals (maximum r_s_ =.61, with .5% of simulated cases above r_s_ >.40). For research labs, we observe a linear tendency that produces stronger correlation coefficients at more restrictive RSSI ranges, reaching a maximum correlation coefficient of r_s_ =.34 at the RSSI interval [-53:-58], with .5% of simulated cases above r_s_ >.19. It is clear that the overall strength of the correlation is much weaker for research labs when compared to the university context or even the private company. Advice-seeking ties for the private company strongly converge for most RSSI ranges, (r_s_ >.30) with a maximum correlation coefficients of r_s_ =.46 at a low range interval [-53:-60], with 2.5% of simulated cases above r_s_ >.36. Similar to friendship ties, advice-seeking ties in the private company context lack a clear linear tendency. Furthermore, we observe that strong advice-seeking ties (4^th^ quartile) generate on average more BT detections per hour compared to dyads reporting weak advice-seeking ties (1^st^ quartile). The corresponding average BT detections per hour between strong/weak self-reported advice-seeking ties are 19/4 for university-based team member dyads, 56/39 for research labs, and 132/84 BT detections for company-based teams (see Table 1).

**Fig. 6:**
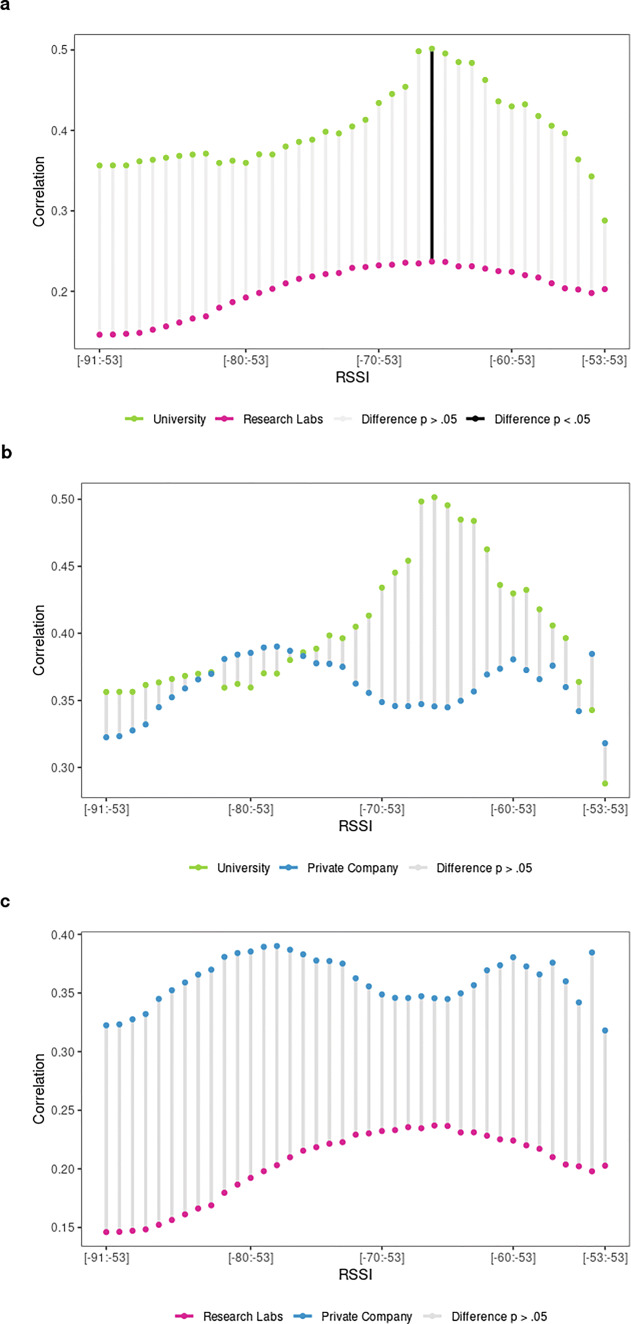
Differences of advice-seeking correlation coefficients at cumulative RSSI intervals comparing organizational contexts

Overall, organizational embedding clearly affects the strength of the convergence for advice-seeking relationships: it increases for university-based teams while tending to be lower for research labs and staying roughly the same for the private company. These differences are marginally significant only when comparing correlation coefficients between university- and lab-based teams using the cocor test (see Fig. 6 and Table 11, 12, 13, in supplementary file S1).

Overall, we can firstly conclude that BT detections converge with both friendship and advice-seeking scores when calculated across all teams. While for both types of social relationships—expressive as well as instrumental ties—the correlation coefficient is larger than .30 and can thus be considered strong, the RSSI threshold to achieve strong correlations differs. While friendship is best captured using almost the entire range of RSSI values [-53:-90], advice-seeking relationships are best captured using a more restrictive set of stronger RSSI values [-53:-66]. The organizational context affects both the overall tendency and the strength of convergence for some groups. These differences between organizational context are, however, only marginally significant. In short, BT detections do converge strongly with both instrumental and expressive ties, while organizational differences do not matter significantly.

### Validity of BT detections at discrete RSSI levels

**Fig. 7:**
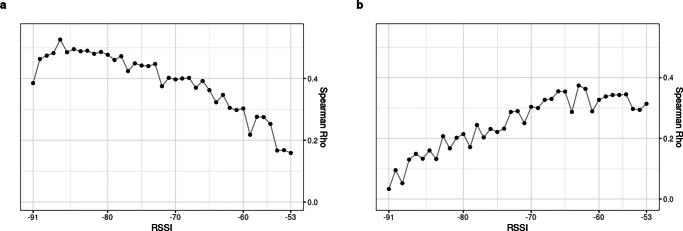
Correlation coefficients for **a** friendship and **b** advice-seeking across all teams at discrete RSSI levels

Next, we turn to analyze the correlation coefficients between self-reported measures and BT detections at each discrete RSSI level separately. We aim to establish whether BT detections at certain specific spatial distances are a valid indicator for friendship and advice-seeking ties in research teams. As Fig. 7 suggests, RSSI levels and thus the spatial distance at which BT detections occur, have an effect on friendship correlation coefficients and on advice-seeking relationships. The results displayed confirm the overall tendency observed in the previous section that friendship ties are better captured at lower RSSI levels indicating a greater spatial distance, while advice-seeking ties are best measured at higher RSSI levels, indicating closer spatial proximity.

Considering the correlation between BT and friendship scores in Fig. 7a first, we can see that these are highest at lower RSSI values (maximum r_s_ =.53 at RSSI = -87. See Table 6 in supplementary file S1). In other words, as the strength of the RSSI signal increases, the frequency of BT detections corresponds less to the actual self-reported friendship scores. In fact, Fig. 7a suggests a linear relationship between RSSI levels and the correlation coefficients, suggesting that friends are more frequently found at a greater spatial distance from each other. The variance explained is 27%. Comparing furthermore the median RSSI level between the group of team members indicating strong friendship ties (4^th^ quartile) versus those indicating weak friendship ties (1^st^ quartile) we observe a RSSI_median_ = -78 for the former and RSSI_median_ = -80 for the latter (see Table 1). Although BT detections occur more frequently among team members with strong friendship ties, spatial distance only plays a moderate role in differentiating the strong versus weak friendship dyads.

However, the situation is clearly different when examining advice-seeking in Fig. 7b, where an inverted linear trend is observable compared to friendship (see Table 7 in supplementary file S1). A maximum correlation is reached at a lower RSSI levels, indicating closer spatial proximity (maximum r_s_ =.37 at RSSI = -63). Advice-seeking relationships are best identified within research teams in situations of close spatial proximity between BT devices. According to Hemphill (2003), the correlation for some higher RSSI values is still strong (r_s_ >.30), but does not reach the same peak correlations above r_s_ >.40 when compared to friendship correlations. The variance explained for advice-seeking is 14%. In addition, we observe a difference in terms of the median RSSI value between strong advice-seeking ties (4^th^ quartile, RSSI_median_ = -76) and weak advice-seeking ties (1^st^ quartile, RSSI_median_ = -80). Team members frequently seeking advice from each other not only generate more BT detections on average, but also tend to be found in greater spatial proximity to each other compared to team members who indicate weaker advice-seeking ties.

Considering BT detections across all teams at each RSSI level separately shows that a greater spatial distance discriminates to some extend friends from non-friends. For advice-seeking, the inverse holds true: closer spatial proximity discriminates better the advice-seeking relationships among team members.

#### Validity of BT detections at discrete RSSI levels and organizational context

**Fig. 8:**
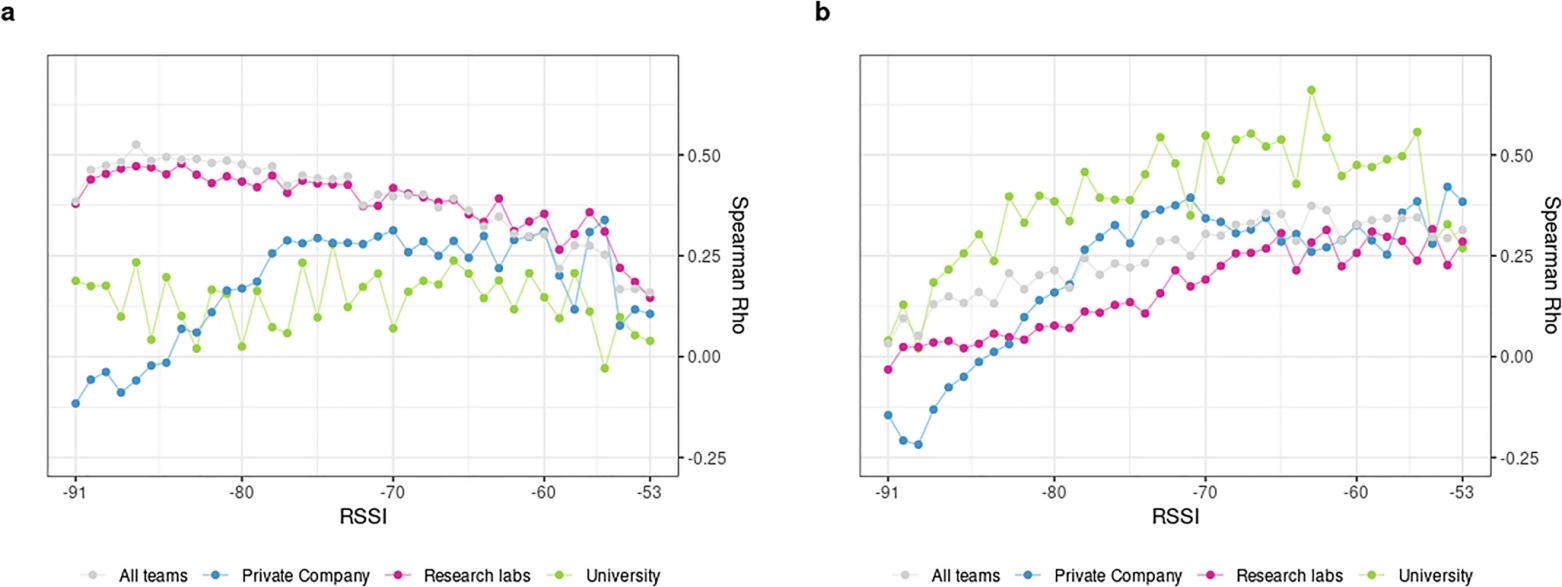
Correlation coefficients of **a** friendship and **b** advice-seeking scores with BT detections at discrete RSSI levels by organizational context

**Fig. 9:**
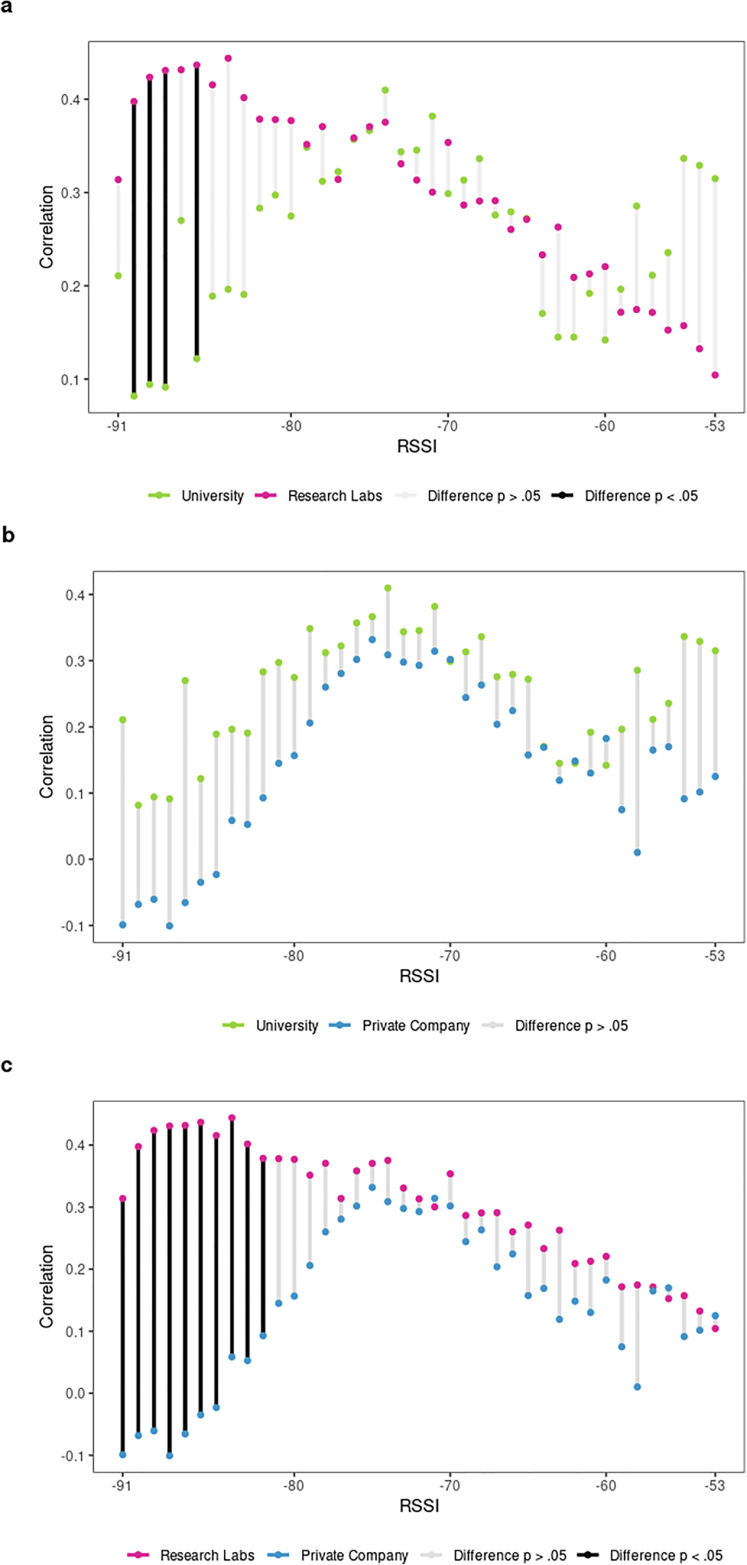
Differences of friendship correlation coefficients at discrete RSSI levels comparing organizational contexts

**Fig. 10:**
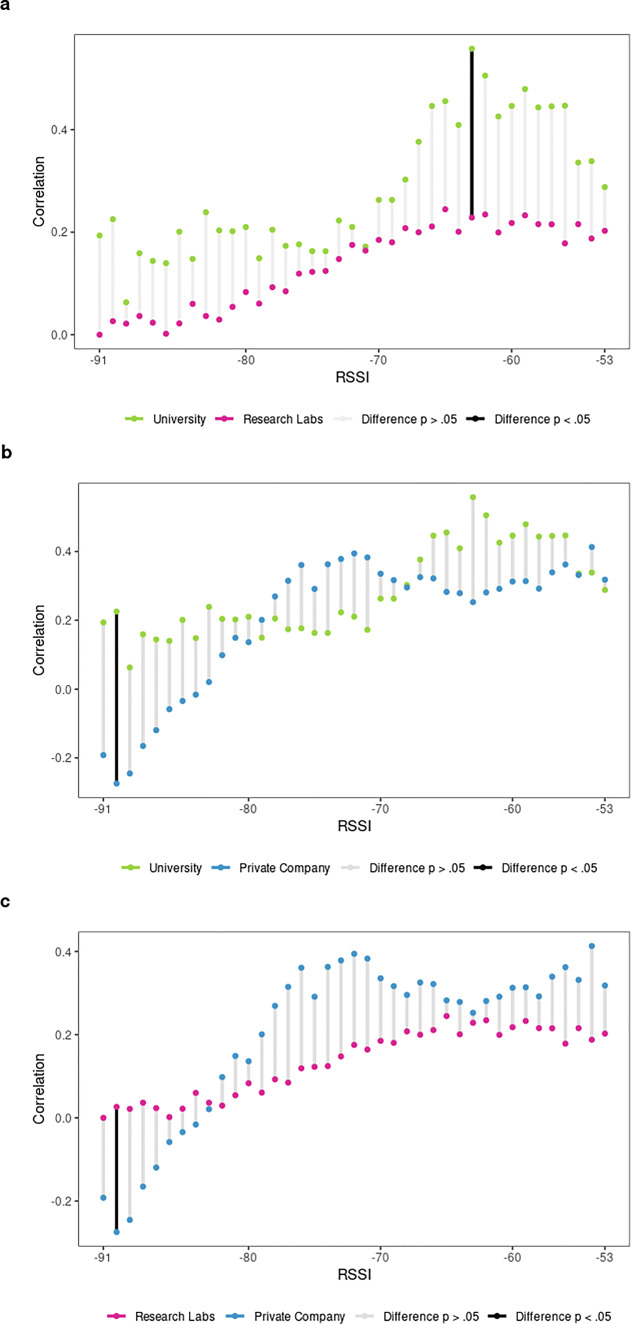
Differences of advice-seeking correlation coefficients at discrete RSSI levels comparing organizational contexts

Next we address how organizational contexts affect the discriminant validity of BT detections (Figs. 8, 9 and 10).

Examining first the friendship ties in Fig. 8a, it is evident that research labs follow the overall tendency to best identify friendship at lower RSSI signals, indicating a greater spatial separation. However, comparing strong (4^th^ quartile) versus weak (1^st^ quartile) friendship ties in research labs indicates that spatial distance only discriminates weakly among expressive ties, with respective RSSI_median_ values of -80 and -79. The overall strength of the correlation decreases for university-based teams (usually r_s_ <.30), while no clear increasing (or decreasing) trend is visible. Thus, for universities we only observe a moderate correlation between BT detections and friendship scores in some isolated instances at best, while it does not appear that spatial proximity has any effect on this relationship. Similar to research labs, strong (4^th^ quartile) versus weak (1^st^ quartile) friendship ties tend to occur roughly at the same spatial distance with corresponding RSSI_median_ values of -74 and -73 respectively. The private company behaves slightly differently again: there appears to be a linear tendency towards stronger correlational coefficients as RSSI values increase from -91 to -79. However, the fact that the correlation coefficients include 0 implies that there is only a weak or no correlation at all between friendship ties and BT detections within the given RSSI range. Correlation coefficients stay moderate (r_s_ <.30) for most of the higher RSSI values, while decreasing considerably at lower RSSI levels, suggesting that in private companies friends are found at close and medium-range distances from each other. Strong (4^th^ quartile) versus weak (1^st^ quartile) friendship ties occur at RSSI_median_ values of -77 and -81 respectively, indicating a somewhat stronger effect of spatial distance on friendship ties. In the private company, high friendship ties are found at closer spatial proximity while weak friendship ties tend to occur at greater spatial distance. The simulated reference distributions for university based as well as private company-based teams furthermore indicate that the observed correlation coefficients could have occurred by chance (see supplementary file S3—Illustration 8 and 10 respectively). For research labs, however, correlation coefficients above r_s_ >.33 are significant, since the chance to observe a correlation as strong only occurs in .5% of all simulated cases. Having knowledge of the spatial distance at which BT detections happen thus primarily improves our ability to identify friends within teams in research labs, while it affects university- or company-based teams to a far lesser extent. The aforementioned differences are significant for research labs versus university-based teams (Fig. 9a, see also S1 - Table 14) and research labs versus the private company at lower RSSI values (Fig. 9c, see also S1 - Table 16)

Considering the advice-seeking ties in Fig. 8b, the overall tendency for instrumental relationships to occur at higher RSSI values indicating closer spatial proximity is remarkable. Although the strength of the correlation is affected by the organizational context, the overall tendency of instrumental ties implying spatial proximity is clearly visible for universities, research labs and the private company. The relationship is strongest for the university context, where the correlation coefficients increase in a linear fashion as RSSI values decrease from r_s_ =.04 at RSSI = -91 to a peak correlation of r_s_ =.66 at RSSI = -63. Comparing strong (4^th^ quartile, RSSI_median_ = -72) versus weak (1^st^ quartile, RSSI_median_ = -84) advice-seeking dyads in universities indicates that spatial distance is crucial for instrumental ties: the observed difference of 12 RSSI levels is the largest difference observed in our study. Advice-seeking clearly involves relations of closer physical proximity in universities. For research labs we observe a similar linear tendency as RSSI values increase. However, the overall strength of the correlation is weak for most RSSI levels (r_s_ <.20 for RSSI < -69), reaching only half of the peak correlation of university-based teams (r_s_ =.32) in isolated instances. The difference between median RSSI values is much smaller in research labs as it is in universities: strong (4^th^ quartile) advice-seeking dyads generate a RSSI_median_ = -77 compared to a RSSI_median_ = -80 for weak (1^st^ quartile) advice-seeking dyads. The private company again exhibits a somehow different pattern in that negative correlation coefficients are observed at lower RSSI values. At a greater spatial separation, high BT detections indicate weak advice-seeking ties (r_s_ = -.22 at RSSI = -89; r_s_ = -.21 at RSSI = -90). Although a linear tendency of increasing correlation coefficients is observed for RSSI values ranging from -88 to -79, the coefficients are weak or even zero, indicating no consistent relationship between BT counts and advice-seeking scores. Comparing the median RSSI of weak (1^st^ quartile, RSSI_median_ = -79) versus strong (4^th^ quartile, RSSI_median_ = -76) advice-seeking ties suggests that spatial distance discriminates to some extend both groups within the context of the private company. For university and private company-based teams we can consider correlation coefficients above r_s_ >.46 as extremely unlikely to occur by chance (.5% of simulated cases), while this threshold is somewhat lower for research labs with r_s_ >.19 (see S1—Table 3). The cocor test for advice-seeking correlations across organizational contexts shows significant differences at a single RSSI level for each conducted comparison (see Fig. 10 and S1 - Table 18).

## Discussion

Given the results of the correlation coefficients for cumulative BT detections, we observe a strong convergence with friendship scores (r_s_ =.50), as well as advice-seeking scores (r_s_ =.42). Our findings suggest that the selection of an arbitrary and/or more restricted range of RSSI will produce sub-optimal results. Rather, RSSI ranges should be carefully selected not only taking into account the type of social relationship but also considering the organizational context. Friendship ties are best identified using all BT detections occurring across the entire range of RSSI levels, whereas advice-seeking relationships converge better with BT detections using a more restrictive RSSI range. Following the proposed heuristic for advice-seeking relationships holds across all three organizational contexts: correlation coefficients are at their respective maximum at a stronger and narrow range of RSSI levels for the university, research lab and company-based teams. However, for friendship ties, university-based teams break with the general pattern of yielding the highest correlation coefficients across all RSSI levels: contrary to research labs or the private company, friendship ties are best captured at more restrictive RSSI ranges within universities.

The role of organizational context is of less importance for cumulative BT detections. However, when examining the validity of BT detections at discrete RSSI levels and specifically the special case of university-based teams, organizational context clearly matters. Using data across all teams, we observe that considering each discrete RSSI level separately improves our ability to identify friendship ties, especially when counting BT detections at lower RSSI values, indicating a greater spatial separation. This means that friends within teams are usually found at a greater spatial distance to each other. The opposite holds for advice-seeking relationships, which have a tendency to occur more frequently at higher RSSI values indicating closer spatial proximity. We can infer that advice-seeking behavior, especially in university-based teams, happens in close spatial encounters, such as when people sit together around a table or computer screen for example. Our empirical results are thus diametrically opposed to the findings reported in Matusik et al., (2018), who “see the strongest convergence between BT detections and reported friendship at RSSI levels that suggest close spatial proximity situations. In contrast, convergence of BT detections with reported advice-receiving and giving relationships was highest at more liberal RSSIs, indicating a greater spatial separation” (Matusik et al.,, 2018, 16). Unfortunately, the reasons that could explain the differences between our findings and those of Matusik et al., (2018) remain largely speculative. Although we observe a larger team size (*n* = 32) in Matusiks’ study compared to our own (*n* ˜ 10), other important information such as the layout of the working environment is not available, making it impossible to compare the results across both studies in a meaningful manner. Thus, a first important contribution of our work therefore indicates that the ability to identify friends or advice-seeking relationships based on the spatial distance at which interactions occur is not consistent across R&D groups. Our ability to identify expressive or instrumental ties with Sociometric badges or BT sensors varies according to other contextual factors that need careful consideration.

Our research design involving multiple teams allows this issue to be further addressed by examining how the organizational context affects these characteristic interaction patterns. As suggested, different organizational arrangements among universities, research labs and the private company restrict our ability to discriminate between different types of relationships at work. Considering advice-seeking relationships first, we note that the strongest correlation coefficient of the entire study (maximum r_s_ =.66) occurs in universities, being markedly different from the more moderate correlation coefficients for research labs (maximum r_s_ =.32) or the private company (maximum r_s_ =.42). It seems that the university context is particularly suitable for identifying instrumental ties among team members. This is not surprising given the fact that university-based teams usually have fewer opportunities to interact by chance while they still have to address work matters. In fact, traditional, cellular office spaces have been consistently identified as inhibiting chance encounters the further away people are located from each other (Sailer and McCulloh, 2012; Toker & Gray, 2008). Furthermore, given the scarce temporal overlap due to teaching responsibilities running in parallel to research activities, random coincidences among university academics are rare. As a consequence, people have to actively seek out the colleagues they want to meet, which in many cases will mirror precisely work related needs. The fact that BT detections are predominantly generated during those instrumental and intentional encounters in close spatial proximity aligns them strongly with the pattern of self-reported advice-seeking ties. Given the working arrangements in universities, other types of encounters which could produce a similar and hence confounding BT proximity pattern are unlikely to occur. In other words, BT detections at high RSSI levels are specific to a single type of social relationship, namely advice-seeking meetings in close spatial proximity.

The situation is different for advice-seeking relationships in research labs or the private company, where team members share office or laboratory space. Advice-seeking relationships within these non-university contexts are part of the overall interaction patterns naturally occurring during the day in a shared working space. As the meaning of being at a close spatial distance to other team members is likely to shift continuously—involving situations of advice-seeking for example, but also other types of encounters such as sharing of laboratory benches—spatial proximity is much less indicative for a single type of social relationship. On the contrary: BT detections now capture a whole variety of encounters, some of which could be interpreted as false positive detections with regards to the self-reported advice-seeking. For example, two research assistants could be working in close proximity to each other not because they need each other’s advice but because the equipment required for their work stands side by side in the laboratory. Indeed, studies regarding the behavioral impact of shared workspaces have shown repeatedly that chance encounters become much more frequent in open office environments (Boutellier et al., 2008; Khazanchi et al., 2018). In open laboratory environments shared experimental equipment has been identified as a particular strong facilitator of chance encounters as people often coincide around them, exchange technical know-how or wait their turn to use them (Andereggen et al., 2013; Heinzen et al., 2018). That is to say, that proximity detections in shared workspace environments hardly follow a single or unique rationale but happen to some degree at random. Since purposeful, close-range detections occur as part of a much larger stream of different types of (random) co-location detections, the overall correlation coefficient for research labs and the private company are clearly weaker. Considering lower RSSI values, however, notable differences between research labs and the private company start to emerge. While the correlation coefficients tend towards zero for research labs, it becomes negative for private company-based teams, indicating that BT detections now coincide consistently with lowest advice-seeking ties. Distant devices are detected equally in both situations, given the shared work environment. However, whereas in the private company, the greater spatial distance of low advice-seeking dyads is more consistent due to the fixed seating layout, in research labs the spatial configuration is much less stable probably due to the mobility of researchers within the laboratory. As patterns of proximity and distance shift continuously, the correlation coefficients tends towards zero for laboratory-based teams, especially as devices at greater spatial distance are registered.

Discussing friendship relationships next, what requires an explanation is the relatively high correlation coefficient for research labs compared to the lower coefficient for university and private company-based teams. Friendship ties are best identified at a greater spatial distance for research labs, whereas this does not hold for teams working at the university or the private company. As the cocor test has shown, these differences between research labs and university-based teams, and between research labs and the private company are statistically significant. The difference is especially puzzling when comparing research labs and the private company, since in both contexts, team members do share laboratory and office space. As suggested previously (see supplementary file S3 Illustration 2), a drop in the correlation coefficient at identical RSSI levels (e.g., -90) can be understood in terms of varying tie strengths for similar spatial configurations and BT detection patterns. Thus, in a laboratory environment, friends are easily detected ‘at a distance’ (i.e., lower RSSI levels) as they share the same work environment. However, at the same time it seems that the colleagues that work in close spatial proximity are mainly those that indicate to have weak friendship ties. Hence, we encounter a particular situation where friends are found at greater spatial distance while non-friends work side by side. This suggests, that proximity among team member dyads follows instrumental rather than expressive necessities: being mobile in the laboratory, it may well be that proximity among team members is conditioned by shared laboratory equipment as mentioned earlier, or senior—junior relations that are independent of actual friendship ties. In addition to facilitating chance encounters, open workspaces have also been identified along these lines as suppressing social conversations due to the lack of privacy (Fayard & Weeks, 2007; Heinzen et al., 2018). Open office or laboratory spaces that are shared among many does not provide the opportunity to engage in more personal or confidential conversations, which are rather reserved for specific times and places such as lunch or coffee breaks (Van Marrewijk & Van den Ende, 2018; Weijs-Perrée et al., 2019). Thus, a higher correlation coefficient between friendship ties at greater spatial distance seems plausible for laboratory environments, as friends are co-present to each other within the lab while detections at closer spatial proximity are governed by chance encounters and operational needs. In short, in a shared laboratory space, a high correlation coefficient is thus not only the expression of friendship ties being detected at a distance but equally the product of non-friends working in close spatial proximity while actual friends meet comparatively seldom. Note the difference in the private company which is exactly the opposite: the further away team members are seated from each other the less they indicate mutual friendship ties. Friendship ties, it seems, mirror closely the seating order where desk neighbors indicate strong friendship ties compared to those who are seated further away. The fixed seating order would also explain to some degree the negative correlation coefficient at greater spatial distance, indicating that this pattern appears rather stable: weak friendship ties are mainly detected at a distance and stronger friendship ties in greater proximity.

An important result emerging from our cross-organizational analysis is the fact that physical proximity is far from being an unequivocal indicator of expressive or instrumental relations. Importantly, it is the wider organizational context that can have a substantial influence on the fit between physical proximity and a certain type of social relationship, as we have seen. This insight echoes the existing research on wearable sensor devices. As others have argued, inferring the friendship network structure through BT signals can be vastly improved when contextual information on work and leisure times and places is taken into consideration (Eagle et al., 2009). As our research design across organizational contexts shows, the validity of BT detections is improved if the organizational environment limits the social situations during which physical co-presence occurs. The distinct feature of university-based teams is precisely that BT detections are predominantly generated during instrumental meetings or not at all. However, in situations of continued co-presence such as labs or shared office spaces, correlation coefficients are considerably weaker as spatial proximity is not just the product of specific instrumental or expressive situations but occurring naturally during many social (and random) situations over the day. Together with our insights into the influence of the organizational environment on interaction patterns, one can begin to formulate some recommendations on the design of research involving wearable sensors; others have provided practical recommendations on field logistics (Chaffin et al., 2017; Kayhan et al., 2018; Parker et al., 2018) and ethical issues (Metcalf & Crawford, 2016; Stopczynski et al., 2014) for wearable sensors.

### Recommendations and limitations

A key recommendation concerns the unavoidable gap that exists between the physical signal (such as RSSI in the case of BT) and the social or psychological constructs it should indicate. Most research using wearable sensors has so far concentrated on the validity of a certain sensor measurement in relation to one or two specific higher-level constructs. However, when assessing the evidence across the emerging body of research on wearable sensors, it is clear that a single physical measurement cannot be an equally valid indicator for such diverse concepts as “happiness”, “friendship”, “advice” or “personality”. As a consequence, researchers need to address the gap that separates the physical measurement from social or psychological constructs and consider opportunities such as mixed methods in their research design in order to bridge it (Müller et al., 2019).

Improving the fit between physical measurement and higher-level construct can involve several steps. A crucial issue is to gain a deeper understanding of when the data points generated by sensors represent and are indeed specific to the desired, socially meaningful units of analysis. Our example of advice-seeking relationships across different organizational environments nicely illustrates this point: the interactions detected in research labs are to a large degree the result of the shared laboratory work space among team members, producing high BT detections irrespective of any substantial (advice-seeking) relationship taking place. In contrast, the scarce interaction opportunities within university teams make interactions socially significant—as advice-seeking relationships in our case. Whereas the BT detections in the former are an artifact of the workplace layout, in the latter they are a more faithful representation of genuine social relationships of interest. Our cautionary note here echoes similar concerns raised in social network research, where it has been shown that the “social organization (social context) and spatial arrangement of the room account for the social relationships formed there” (Doreian & Conti, 2012). Thus, organizational scholars should think carefully about the contextual markers that allow the identification of conceptually meaningful relationships. As Kozlowski and Chao (2018) argue, a data pattern that qualifies an interaction as such is dependent on many contextual variables and the focus of the research. It is influenced not only by spatial arrangements but also by different temporal scales, comparing for example interactions in an emergency department versus interaction patterns on a space exploration mission to Mars. As there are many pathways that lead to an interaction, it is the task of the researcher to construct “flexible inferential frameworks” which are sensitive to these contextual differences and identify conceptually relevant interactions in general and sensor data in particular. Coming back to the case study presented here, in the case of advice-seeking relationships in research labs, this might involve the identification of small group meeting patterns that distinguish advice-seeking encounters from the overall, spatially conditioned BT deluge. In some cases, this might well lead to the conclusion that wearable sensors are not suited to the measurement of specific constructs under certain organizational conditions.

As with other studies, this research has important limitations. First, we have examined only one contextual variable in terms of its effect on the validity of BT data. Other important variables to be explored include team tenure or team size. Interaction patterns are likely to be influenced by the familiarity of team members with each other. Similarly, as the opportunities for different advice-seeking partners grow in larger teams, advice-seeking behavior might be more strongly influenced by friendship preferences. However, our current analytical approach based on an examination of the correlation coefficients between two groups makes the examination of more than one contextual variable cumbersome. Other analytical techniques should be used.

A second limitation concerns the 5-day period over which interactions were collected. A longer period of data recollection is probably desirable to more reliably capture the overall interaction patterns among team members. To the best of our knowledge, there is currently no published study available that would allow the day-to-day variability of interaction patterns to be assessed. Matusik et al., (2018) go some way in this direction, showing that a “great deal of variance” (p.23) is associated with daily networks, but do not provide any criteria for a closer examination. Monitoring interaction patterns in research groups over several weeks or even months and analyzing the temporal scale at which these patterns stabilize would provide an interesting research opportunity.

A third limitation concerns the relatively few R&D teams for each organizational setting. Only three university groups were compared with three teams in a private company and three teams in research labs. This is certainly a limitation in terms of the variety of team settings and environments, even within each of the organizational settings. Including more teams in future studies would also allow us to go beyond the influence of organizational variables on the validity of BT detections. Age, gender, available space, type of encounter or cultural background are all known factors to affect the personal space/distance during interactions (Knapp et al.,, 2014, p. 133). Thus, it remains an open question to which degree BT measures correlate with self-reported ties when teams across different cultures are compared. Individuals from “contact cultures” (e.g., countries in the Mediterranean area) tend to require a smaller personal space during interactions than individuals from “non-contact cultures” (e.g., countries from Northern Europe) which in turn could influence the frequency of BT measures at certain RSSI levels (Andersen et al., 2013; Beaulieu, 2004; Hall, 1992). Although our sample of research groups includes teams from Spain and the UK, the overall number of participating teams is too small as to make any generalizable findings. However, our results nevertheless raise awareness of the context-sensitive nature of wearable sensor measurements. Further studies are sure to clarify the extent to which our findings are representative for certain organizational environments at large and allow the role of contextual factors in the validity of BT measurements to be further substantiated.

## Conclusions

Our study contributes to the emerging research on the validity of wearable sensor measures for organizational research. We have shown first of all that instrumental and expressive ties converge considerably with BT detections; we also provide evidence on the importance of spatial proximity regarding the validity of BT detections at discrete RSSI levels. Furthermore, our research indicates the influence of the organizational context on the validity of BT data for identifying friendship and advice-seeking relations across 9 small R&D teams. The organizational context matters when analyzing sensor data in terms of the strength of the observed correlation coefficients, as well as distinguishing between friendships and advice-seeking in relation to spatial proximity. Advice-seeking ties are best identified in close spatial proximity, especially in the university context. Friendship ties, on the other hand, are best identified at a greater spatial distance for research labs primarily. Given the general importance of the organizational context with regard to the meaning and interpretation of social phenomena, our findings should be extended beyond proximity measures to other dimensions of sensor data such as accelerometers or audio measurements. Complementary data and mixed-methods need to be deployed in order to take advantage of high-resolution behavioral data. We hope that this article will contribute to the consolidation of wearable sensors as exciting new research instruments for the social sciences, driving forward the exploration of dynamic group processes in particular.

## Footnotes

1 “Sociometric” (capital “S”) refers here to “Sociometric badges”, the wearable sensor devices used in our research and produced by Humanyze (Boston, USA), formerly called “Sociometric Solutions”. We use “sociometric” (lowercase “s”) when referring to the broader methods and data regarding the study of social relationships.
